# Optimizing high-resolution knee MRI at 3 tesla: conventional acceleration versus deep learning reconstruction

**DOI:** 10.1186/s12880-026-02251-0

**Published:** 2026-03-03

**Authors:** Dominik Deppe, Markus Herbert Lerchbaumer, Khalid M. Baghdadi, Hassan Ali Alyousef, Leila Vivien Nitschke, David Kohnert, Dominik Geisel, Andreas Pohlmann, Moritz Wagner, Thula Walter-Rittel

**Affiliations:** 1 Department of Radiology, Charité - Universitätsmedizin Berlin, Charitéplatz 1, 10117 Berlin, Germany; 2 Siemens Healthineers AG., Berlin, Germany

**Keywords:** Deep learning, Artificial intelligence, Magnetic resonance imaging, Knee

## Abstract

**Background:**

To compare subjective and objective image quality and resolution between time-optimized standard knee MRI (sMRI) with image quality-optimized DL-enhanced (DL-MRI) at 3 Tesla.

**Methods:**

A retrospective single-centre study of 150 knee MRI examinations (75 sMRI, 75 DL-MRI) was conducted. Protocols included Proton density–weighted sequence with fat suppression (PD-FS) (coronal/sagittal/axial), T1 (coronal or sagittal), and T2 (axial) optimized for time in sMRI and for image quality in DL-MRI. Three blinded readers with different levels of experience rated overall image quality, anatomical delineation, fat saturation, motion artefacts, and foreign-body artefacts on 5-point Likert scales. Quantitative analysis was performed to calculate SNR, CNR, and generalised metrics (gSNR, gCNR). Group differences were assessed using two-sided Welch’s t-tests.

**Results:**

Readers rated DL higher in nearly all categories and sequences, with mean gains of ~ 0.48–0.70 for overall image quality and ~ 0.38–0.54 for anatomical delineation (all *p* ≤ 0.001). Fat saturation improved for PD-FS coronal and axial, motion artefacts improved for PD-FS coronal and sagittal (and slightly for axial), and foreign-body artefacts were comparable. Quantitatively, PD-FS showed higher muscle SNR and higher gSNR/gCNR with DL (bone SNR non-significant); T2 showed higher bone SNR and higher CNR/gCNR but lower muscle SNR/gSNR; and T1 showed lower SNR/gSNR with preserved CNR. Compared with sMRI, DL-MRI achieved a twofold improvement in in-plane resolution (0.4 × 0.4 mm² vs. 0.2 × 0.2 mm²) and reduced slice thickness (3.0 mm vs. 2.5 mm. and down to 1.0 mm for T2-weighted sequences), while slightly shortening total scan time (10:14 min vs. 9:30 min).

**Conclusion:**

DL-MRI provided superior image quality and higher resolution over time-optimized standard knee MRI at 3 Tesla.

**Trial registration:**

Not applicable.

**Supplementary Information:**

The online version contains supplementary material available at 10.1186/s12880-026-02251-0.

## Introduction

Magnetic resonance imaging (MRI) is an essential imaging modality for the evaluation of musculoskeletal (MSK) diseases, particularly in the depiction of complex anatomical structures like the knee joint. High-resolution imaging is necessary to assess cartilage, ligaments, menisci, and the bone accurately. However, achieving high spatial resolution in conventional MRI protocols typically requires long acquisition times, which can increase patient discomfort, motion artefacts, and reduce scanner throughput.

Deep learning (DL)–based reconstruction has recently emerged as a powerful technique to enhance image quality and spatial resolution without the need for extended scan times. DL-MRI relies on convolutional neural networks (CNNs) trained on large datasets of fully sampled and undersampled MRI scans [1, 2]. These models learn to predict high-quality images from sparsely sampled input data, enabling faster acquisitions [3]. DL reconstruction can denoise [4, 5], reduce motion artefacts [6], and enhance image sharpness [7] in a data-driven manner, often outperforming classical algorithms in both speed and perceptual quality [3]. However, conventional MRI imaging protocols also can be accelerated by lowering spatial resolution, decreasing the number of signal averages, using partial Fourier or parallel imaging with high acceleration factors. While these approaches reduce acquisition time, they typically come at the expense of signal-to-noise ratio (SNR), artefact suppression, and overall image sharpness.

In knee-MRI, DL-enhanced protocols have shown significant promise [8]. Recent studies using DL reconstructions can match or surpass the quality of conventional reconstructions while reducing scan time by up to 75% [9]. Moreover, recent research has confirmed that DL-based reconstructions improve image quality in key MSK sequences such as proton-density-weighted (PD) fast spin echo with fat saturation (FS) and high-resolution T2-weighted imaging, both of which are essential for knee joint evaluation [10].

This retrospective study aimed to compare time-optimized conventional knee MRI with image quality-optimized DL-enhanced sequences at 3 Tesla in terms of resolution, subjective assessment, and objective image quality.

## Methods

### Patients

This retrospective single-centre study included patients referred for clinical knee MRI between 03.01.2024 and 07.06.2025. Eligible participants were adults aged over 18 years with a complete MRI dataset available for evaluation. Exclusion criteria included patients under 18 years of age and those with incomplete imaging data. The study was approved by the local ethics committee (EA1/196/25).

### Imaging protocol

All patients underwent 3 Tesla MRI of the knee. Patients underwent conventional standard MRI (sMRI) at 3T (Siemens Magnetom Vida. Syngo MR XA50. Siemens Healthineers) using a time-optimized standard-protocol or image quality-optimized DL-enhanced protocol (DL-MRI) at 3T (Siemens Magnetom Vida. Syngo MR XA60. Siemens Healthineers), using “Deep Resolve Boost” and “Deep Resolve Sharp”. Outpatient were assignment to DL-MRI or sMRI primarily based on scanner availability and scheduling, while inpatients predominantly were scanned using the standard protocol. The evaluated sequences included: PD Turbo spin echo (TSE) fat-saturated (FS) in coronal, sagittal and axial orientation as well as T1 in coronal or sagittal orientation and HR T2 in axial orientation. Scan information (e.g., scan time) were extracted from the protocol configurations on the MRI console. Further details can be found in Table 1.

**Table 1:** Acquisition parameters for the standard and deep-learning MRI protocols for PD-FS (coronal/sagittal/axial), T1 (coronal/sagittal), and T2 (axial) sequences

	sMRI					DL-MRI				
	PD FS coronal	PD FS sagittal	PD FS axial	T1 coronal / sagittal	T2 axial	PD FS coronal	PD FS sagittal	PD FS axial	T1 coronal / sagittal	T2 axial
Reconstructed Voxel	0.4 × 0.4	0.4 × 0.4	0.4 × 0.4	0.4 × 0.4	0.2 × 0.2	0.2 × 0.2	0.2 × 0.2	0.2 × 0.2	0.2 × 0.2	0.2 × 0.2
Field of View Read (in mm)	180	160	150	160	140	180	160	150	160	140
Field of View Phase	87.5%	100%	100%	100%	100%	87.5%	100%	100%	100%	100%
Oversampling	30%	100%	50%	100%	80%	30%	100%	60%	100%	80%
Averages	1	1	1	1	1	1	1	1	1	1
Flip Angle	15°	140°	150°	140°	150°	15°	140°	150°	140°	150°
Base Resolution	384	448	400	448	400	352	368	352	385	352
Phase Resolution	80%	60%	70%	60%	70%	85%	90%	90%	90%	100%
Interpolation	Off	Off	Off	Off	On	On	On	On	On	On
Slice thickness	3.0	3.0	3.0	3.0	1.0	2.5	2.5	2.5	2.5	1.0
Number of slices	32	31	31	35	35	33	31	35	33	35
TR (in ms)	3540	3320	3320	729	6120	4000	3300	4000	570	3800
TE (in ms)	36	36	36	11	59	29	31	33	11	106
Bandwidth (in Hz/Px)	179	179	179	199	245	246	240	230	246	249
Turbo factor	7	7	9	4	17	7	7	7	3	14
Number of signal averages	1	1	1	1	1	1	1	1	1	1
Acceleration factor	GRAPPA 2	GRAPPA 2	GRAPPA 2	GRAPPA 2	GRAPPA 2	GRAPPA 4	GRAPPA 4	GRAPPA 4	GRAPPA 4	GRAPPA 4
Scan time	1:55 min	2:01 min	1:54 min	2:20 min	2:04 min	1:38 min	1:45 min	2:00 min	1:44 min	2:23 min
Total scan time:	**10:14 min**					**9:30 min**				

### Subjective image quality assessment

Image quality was independently rated by three radiologists: a radiologist with < 1 year expertise in musculoskeletal imaging (beginner); a radiologist with 4 years of expertise in musculoskeletal imaging (intermediate) and an expert in musculoskeletal imaging with 8 years expertise (expert). All readers rated imaging datasets blinded for scanner type and all patient information including sex, age, clinical information and final diagnosis using a 5-point Likert scale for overall image quality [1–5]. Delineation of cartilage, muscle, ligament contour / meniscal contour and tendon contour [1–5], fat saturation [1–5], artefacts caused by motion [1–5] and artefacts caused by foreign body [1–5]. All details for the 5-point Likert scale can be found in Table 2. All readers rated in consensus two additional cases before individual scoring to optimize interrater reliability.

**Table 2:** Scoring scheme. Five-point Likert scale [1–5] used to rate overall image quality, anatomical delineation, fat saturation, and artefacts, ranging from 1 = non-diagnostic/severe impairment to 5 = excellent/no impairment

	1	2	3	4	5
Overall image quality	Non-diagnostic	Poor but somewhat diagnostic	Acceptable	Good	Excellent
Delineation of Cartilage. Muscle and tendon contour. Ligament contour / meniscal contour	Insufficient delineation of anatomy (reliable diagnostic interpretation not possible)	Very limited delineation of anatomy (partial assessment possible with low diagnostic confidence)	Somewhat impaired delineation of anatomy	Only minimally reduced delineation of anatomy	Excellent delineation of anatomy
Fat saturation	Inhomogeneous and incomplete. Detrimental to diagnostic image information	Inhomogeneous and incomplete; probably detrimental to diagnostic image information	Inhomogeneous and incomplete but likely non-detrimental to diagnostic information	Incomplete but no detrimental to diagnostic information	Complete and homogeneous fat saturation
Artefacts caused by motion	Severe. Non-diagnostic imaging	Pronounced. Detrimental to diagnostic image quality	Relevant. Non-detrimental to diagnostic image quality	Minimal. no relevant impairment to diagnostic image quality	None
Artefacts caused by foreign body	Severe. Non-diagnostic imaging	Pronounced. Detrimental to diagnostic image quality	Relevant. Non-detrimental to diagnostic image quality	Minimal. No relevant impairment to diagnostic image quality	None

### Objective image quality assessment

Quantitative analysis was performed by a fourth independent reader (radiologist with 5 years of experience in musculoskeletal imaging). One circular ROI with a diameter of 10 mm was placed in the central tibial plateau, and one ROI with a diameter of 10 mm was placed in the adjacent muscle (e.g., medial gastrocnemius or vastus medialis). An additional background ROI was used to characterize noise.

For baseline comparison, SNR and CNR were calculated as follows:

- SNR bone= mean signal bone/ background SD.
- SNR muscle= mean signal muscle/ background SD.
- CNR = (mean signal bone – mean signal muscle)/background SD.

Because background-based metrics are limited in DL-reconstructed MRI—where nonlinear denoising alters noise statistics — generalised metrics were also evaluated, reflecting local consistency, as described by Reschke et al. [11] :

- gSNR bone = mean signal bone/ SD bone.
- gSNR muscle = mean signal muscle/ SD muscle.
- gCNR bone = (mean signal bone – mean signal muscle)/ SD bone.
- gCNR muscle = (mean signal bone – mean signal muscle)/ SD muscle.

### Statistics

Statistical analysis was performed using IBM SPSS Statistics (version 31). For each sequence, the mean rating of each image quality criterion was calculated per reader, and the mean across the three readers was computed per patient. Although Likert-scale data are ordinal, averaging across raters allows the use of parametric tests; therefore, computed means per sequence (DL-MRI vs. sMRI) were compared using two-sided Welch’s t-tests.

Quantitative imaging parameters (SNR bone/muscle, gSNR bone/muscle, CNR, and CNR bone/muscle) were assessed for normality using the Shapiro–Wilk test. As all parameters were approximately normally distributed, comparisons between sMRI and DL-MRI for each sequence were performed using two-sided Welch’s t-tests. Means and standard deviations are reported. A p-value < 0.05 was considered statistically significant.

## Results

### Patients

**Table 3:** Clinical indications for MRI of the knee

Clinical indications	Total	sMRI	DL-MRI
Suspected meniscus lesion	30	14	16
Post-trauma	15	11	4
Trochlear dysplasia	1	0	1
Osteoarthritis	15	5	10
Suspected cartilage defects	33	11	22
Non-specific pain	40	28	12
Suspected anterior cruciate ligament injury	14	5	9
Suspected posterior cruciate ligament injury	1	1	0
Suspected collateral ligament injury	1	0	1

A total of 150 patients were included (75 sMRI and 75 DL-MRI). Overall, 66 were female (44.0%), with similar distributions across groups (sMRI: 32/75. 42.7%; DL-MRI: 34/75. 45.3%). The mean age was 41.08 years overall (sMRI: 42.20 years; DL-MRI: 39.96 years). Clinical indications for MRI of the knee can be found in Table 3.

### Imaging protocol

**Fig. 1:**
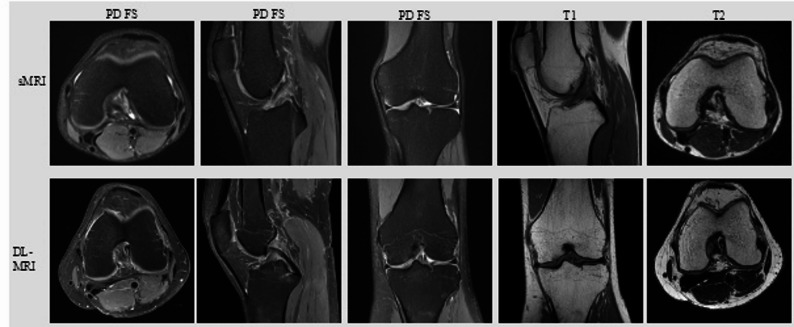
Comparison between standard MRI (sMRI) and deep learning–based MRI (DL-MRI) demonstrates the substantial enhancement of image resolution achieved with DL-MRI

In comparison to sMRI, DL-MRI enabled a twofold improvement in in-plane resolution (reconstructed voxel size 0.4 × 0.4 mm² vs. 0.2 × 0.2 mm²) while simultaneously reducing slice thickness (3.0 mm vs. 2.5 mm) in all three orientations and T1 sequences, whereas T2 sequences were acquired with identical resolution and slice thickness in both protocols. Despite these substantial gains in spatial resolution, the total scan time was slightly reduced (10:14 min for sMRI vs. 9:30 min for DL-MRI, ~ 7% reduction). The number of signal averages was kept constant (NSA = 1) across all sequences, ensuring comparability of acquisition conditions. Despite two longer sequences (PD FS axial and T2 axial), the overall examination time was shorter with DL. An overview of all scan times can be found in Table 2. Example images of both imaging protocols can be found in Fig. 1.

### Subjective image quality assessment

DL-based sequences yielded consistently higher reader scores across most sequences and criteria (see Table 4).

Overall image quality improved by 0.48–0.70 points with DL across PD-FS (coronal, sagittal, axial), T2, and T1 (all *p* ≤ 0.001); the largest gain was seen for T2 (DL-MRI 4.58 ± 0.53 vs. sMRI 3.88 ± 0.61. Δ = 0.70). Anatomical delineation (cartilage, muscle–tendon, ligament/meniscus) was likewise higher with DL for all sequences (Δ 0.38–0.54. all *p* ≤ 0.001). Fat saturation was modestly better with DL for PD-FS coronal (4.43 ± 0.60 vs. 4.28 ± 0.63; *p* = 0.03) and PD-FS axial (4.51 ± 0.57 vs. 4.28 ± 0.64; *p* < 0.001), with no difference in the sagittal plane (*p* = 0.49). Fewer motion artefacts were rated in DL for PD-FS coronal and sagittal (Δ = 0.64 and 0.31, respectively; both *p* ≤ 0.001) and slightly fewer for PD-FS axial (Δ = 0.13; *p* = 0.03), while T1 and T2 showed no difference (*p* ≥ 0.14). Foreign-body artefacts did not differ significantly in any sequence (all *p* ≥ 0.33). Overall, DL-based protocols improved perceived image quality—particularly overall quality and anatomical delineation—while maintaining a similar artifact profile.

Across experience levels — beginner, intermediate, and expert—readers consistently rated the DL-based sequences higher than standard MRI in nearly all qualitative categories, with only isolated metrics showing parity. Full reader-stratified results are provided in Supplement 1.

**Table 4:** Reader-rated image quality (mean ± SD; 5-point Likert) comparing standard MRI (sMRI) and deep-learning MRI (DL-MRI) across PD-FS (coronal/sagittal/axial), T2, and T1, p-values from two-sided Welch’s t-tests

	PD FS coronal			PD FS sagittal			PD FS axial			T2			T1		
	sMRI	DL-MRI	*p*-value	sMRI	DL-MRI	*p*-value	sMRI	DL-MRI	*p*-value	sMRI	DL-MRI	*p*-value	sMRI	DL-MRI	*p*-value
Overall image quality	3.81 (± 0.60)	4.44 (± 0.50)	<0.001*	4.01 (± 0.55)	4.51 (± 0.47)	< 0.001*	3.85 (± 0.54)	4.33 (± 0.67)	< 0.001*	3.88 (± 0.61)	4.58 (± 0.53)	< 0.001*	4.08 (± 0.60)	4.60 (± 0.54)	< 0.001*
Delineation (cartilage/muscle–tendon/ligament–meniscus)	3.89 (± 0.59)	4.43 (± 0.59)	< 0.001*	3.99 (± 0.62)	4.52 (± 0.55)	< 0.001*	3.90 (± 0.62)	4.28 (± 0.73)	< 0.001*	4.07 (± 0.68)	4.56 (± 0.55)	< 0.001*	4.10 (± 0.66)	4.60 (± 0.52)	< 0.001*
Fat saturation	4.28 (± 0.63)	4.43 (± 0.60)	0.03	4.44 (± 0.60)	4.48 (± 0.54)	0.49	4.28 (± 0.64)	4.51 (± 0.57)	< 0.001*	-	-	-	-	-	-
Motion artefacts	4.24 (± 0.63)	4.88 (± 0.33)	< 0.001*	4.49 (± 0.62)	4.80 (± 0.42)	< 0.001*	4.18 (± 0.62)	4.31 (± 0.65)	0.03	4.70 (± 0.59)	4.65 (± 0.57)	0.91	4.81 (± 0.38)	4.76 (± 0.38)	0.14
Foreign-body artefacts	3.30 (± 0.51)	3.54 (± 0.29)	0.49	3.31 (± 0.49)	3.54 (± 0.28)	0.33	1.92 (± 0.90)	3.45 (± 0.32)	0.65	3.48 (± 0.32)	3.67 (± 0.00)	0.53	3.32 (± 0.45)	3.58 (± 0.17)	0.80

### Objective image quality assessment

SNR (bone/muscle), gSNR (bone/muscle), CNR, and gCNR (bone/muscle) were all higher in PD-FS with DL-reconstruction than in sMRI (*p* < 0.05), however, SNR bone did not reach significance (*p* = 0.76). For T2, DL-MRI increased CNR and gCNR (bone/muscle) and raised bone SNR, while gSNR in bone showed no significant difference and muscle SNR/gSNR were lower with DL. In T1, DL-MRI showed lower SNR and gSNR in both tissues; CNR and gCNR (muscle) did not differ significantly, whereas gCNR (bone) was lower with DL. Importantly, these differences were observed despite a doubling of the acceleration factor (from GRAPPA 2 to GRAPPA 4) and a substantially higher spatial resolution in the DL protocol. Further details can be found in Table 5.

**Table 5:** Quantitative image-quality metrics (mean ± SD) comparing standard MRI (sMRI) and deep-learning MRI (DL-MRI) across PD-FS, T2, and T1 (SNR/gSNR/CNR/gCNR); p-values from two-sided Welch’s t-tests

	sMRI (Mean ± SD)	DL-MRI (Mean ± SD)	*p*-value
**PD FS**			
SNR bone	42.21 (± 13.39)	46.28 (± 14.50)	0.08
SNR muscle	98.28 (± 28.54)	135.17 (± 44.38)	< 0.001*
gSNR bone	7.08 (± 2.67)	9.69 (± 2.50)	< 0.001*
gSNR muscle	21.62 (± 26.61)	33.74 (± 10.85)	< 0.001*
CNR	56.07 (± 23.15)	88.88 (± 31.83)	< 0.001*
gCNR bone	9.24 (± 4.04)	18.66 (± 5.76)	< 0.001*
gCNR muscle	12.38 (± 4.68)	22.02 (± 7.22)	< 0.001*
**T2**			
SNR bone	59.69 (± 19.87)	83.91 (± 25.77)	< 0.001*
SNR muscle	21.81 (± 9.14)	14.00 (± 6.26)	< 0.001*
gSNR bone	8.78 (± 2.28)	9.51 (± 3.66)	0.15
gSNR muscle	7.14 (± 2.27)	5.57 (± 1.84)	< 0.001*
CNR	37.88 (± 16.04)	69.91 (± 21.90)	< 0.001*
gCNR bone	5.54 (± 2.26)	7.91 (± 3.08)	< 0.001*
gCNR muscle	12.79 (± 5.41)	28.73 (± 9.17)	< 0.001*
**T1**			
SNR bone	264.27 (± 81.23)	229.98 (± 65.40)	0.005*
SNR muscle	104.32 (± 42.91)	54.49 (± 24.04)	< 0.001*
gSNR bone	23.20 (± 7.94)	13.52 (± 5.31)	< 0.001*
gSNR muscle	23.40 (± 10.56)	11.98 (± 5.06)	< 0.001*
CNR	159.94 (± 80.97)	175.49 (± 53.33)	0.17
gCNR bone	13.85 (± 7.78)	10.22 (± 3.97)	< 0.001*
gCNR muscle	38.60 (± 20.26)	41.02 (± 15.32)	0.41

## Discussion

In this retrospective, single-centre cohort of 150 patient, a DL-enhanced protocol of the knee improved subjective and objective image quality across sequences and improved resolution without prolonging scan time.

In our study, overall quality and anatomical delineation were consistently higher with DL for PD-FS, T2, and T2, with only small sequence-specific exceptions. This improvement is primarily attributable to the higher spatial resolution achieved with DL-MRI, which resulted in more detailed and sharper depiction of anatomical structures.

The subjective gains align with DL’s denoising and edge-preserving properties, as well as its higher spatial resolution, which together can increase perceived sharpness and anatomical conspicuity even when classical SNR decreases in some tissues/sequences. The strong improvements of gSNR/gCNR in PD-FS—and the higher CNR/gCNR in T2—suggest a more homogeneous, locally consistent signal rather than merely a global noise reduction. The lower T1 SNR/gSNR with DL likely reflects the trade-off between aggressive noise suppression/regularization and absolute signal magnitude, while maintaining contrast sufficient for clinical reading (unchanged CNR). The divergence between background-based metrics (SNR/CNR) and generalised metrics (gSNR/gCNR) underscores that DL alters noise statistics; thus. local-consistency measures are more appropriate for DL-reconstructed data. The mixed behaviour of classical SNR with DL observed here has also been described and is attributed to non-Gaussian, spatially varying noise after DL processing [12–14], supporting the use of generalised metrics.

Prior MSK studies reported that DL reconstructions shorten scans and preserve or improve image quality, particularly on PD-weighted FSE/FS and high-resolution T2 sequences [10, 15, 16]. Our findings corroborate these reports: readers across experience levels (beginner, intermediate, expert) favoured DL in nearly all qualitative categories. and the largest subjective gain occurred on T2—sequences where fine texture and low-contrast edges benefit most from DL-driven denoising and sharpening [17, 18]. “Deep Resolve Boost” and “Deep Resolve Sharp” have been reported to improve signal-to-noise ratio and structural delineation while maintaining accuracy in prior phantom studies and clinical evaluations, supporting the methodological validity of these approaches [19, 20]. Nevertheless, rigorous validation remains essential to ensure diagnostic reliability and to exclude potential reconstruction-related biases.

Even in settings with already well-optimized knee MRI protocols and high baseline image quality, such as those established at our centre, DL-based reconstruction provided an additional benefit. The improvements in image quality and workflow efficiency demonstrate that DL is not only valuable for accelerating suboptimal protocols but also enhancing established, high-quality sequences.

This was a retrospective, single-centre study with non-random assignment to protocols and different scanners (of the same make and model), introducing potential selection and hardware/coiling confounds. Acquisition parameters differed between sMRI and DL (e.g., TE/TR, in-plane resolution/slice thickness), so effects cannot be ascribed to the DL algorithm alone.

One main limitation of this work is that we assessed image quality only but not diagnostic accuracy or clinical outcomes. This is due to our retrospective study design and the absence of a gold standard in many patients. As diagnostic accuracy is highly important future studies should include lesion-level sensitivity/specificity. We did not apply semi-quantitative scoring systems (e.g., WORMS), as the study focused on subjective image quality and anatomical delineation rather than lesion assessment. ROI-based quantitative measures may be sensitive to placement and tissue heterogeneity; background-based SNR/CNR are suboptimal for DL data, which is why we complemented them with generalised metrics. A direct comparison in the same patient or a comparison using same parameters was not made in our analysis. Scan times from DICOM headers reflect sequence durations and not room-time or planning overhead. Further time-optimized DL-sequences have the potential of reducing imaging cost while preserving image quality. Time-optimized DL sequences may help reduce imaging costs while maintaining high image quality.

DL-enhanced knee MRI reduced nominal scan time and improved subjective image quality—especially overall quality and anatomical delineation—across readers and sequences. with quantitative gains most pronounced in PD-FS (gSNR/gCNR) and T2 (CNR/gCNR). T1 showed lower classical SNR/gSNR without relevant loss of perceived quality. In our cohort, patients were assigned to either DL-MRI or conventional MRI primarily based on scanner availability rather than clinical characteristics, although a randomized prospective study design would be ideal. Our results support integrating DL reconstructions into routine knee MRI, while prospective, randomized, multicenter studies with harmonized parameters and diagnostic endpoints are needed to confirm generalizability and clinical impact.

In conclusion, even highly optimized knee MRI protocols with excellent baseline quality benefited from DL-based reconstruction. which provided additional gains in image quality and resolution beyond simple acceleration.

## Supplementary Information

Below is the link to the electronic supplementary material.

Supplementary Material 1

## Data Availability

The datasets generated and analysed during the current study are not publicly available due to data protection and privacy regulations but are available from the corresponding author on reasonable request and with appropriate institutional approvals.

## Abbreviations

CNR: Contrast-to-noise ratio
CNN: Convolutional neural network
DL: Deep learning
DL-MRI: Deep learning–enhanced magnetic resonance imaging
EA: Ethics approval
FOV: Field of view
FS: Fat saturation
FSE: Fast spin echo
gCNR: Generalised contrast-to-noise ratio
gSNR: Generalised signal-to-noise ratio
GRAPPA: Generalised Autocalibrating Partial Parallel Acquisition
HR: High resolution
MRI: Magnetic resonance imaging
MSK: Musculoskeletal
NSA: Number of signal averages
PD: Proton density
ROI: Region of interest
SD: Standard deviation
SNR: Signal-to-noise ratio
sMRI: Standard MRI
T1/T2: T1-weighted / T2-weighted sequence
TE: Echo time
TR: Repetition time
TSE: Turbo spin echo
3T: 3 Tesla
