# Supplementary material for: Optimizing high-resolution knee MRI at 3 tesla: conventional acceleration versus deep learning reconstruction
Source: BMC Med Imaging. 2026 Mar 3;26:180. doi: 10.1186/s12880-026-02251-0 (PMC13064314; doi:10.1186/s12880-026-02251-0)
Supplement: Supplementary file 1 — Supplementary Material 1 [file 12880_2026_2251_MOESM1_ESM.docx]

## Supplement:

|  | **PD FS coronal** | | **PD FS sagittal** | | **PD FS axial** | | **T2** | | **T1** | |
| --- | --- | --- | --- | --- | --- | --- | --- | --- | --- | --- |
|  | **sMRI** | **DL-MRI** | **sMRI** | **DL-MRI** | **sMRI** | **DL-MRI** | **sMRI** | **DL-MRI** | **sMRI** | **DL-MRI** |
| **Overall image** **quality** | | | | | | | | | | |
| **Reader 1 (HA)** | 3,80 (±0,57) | 4,77 (±0,45) | 4,03 (±0,43) | 4,77 (±0,42) | 3,73 (±0,50) | 4,31 (±0,73) | 3,37 (±0,56) | 4,60 (±0,55) | 4,15 (±0,46) | 4,69 (±0,57) |
| **Reader 2 (KB)** | 3,77 (±0,56) | 3,97 (±0,52) | 3,96 (±0,53) | 4,08 (±0,49) | 3,91 (±0,50) | 4,00 (±0,59) | 4,12 (±0,64) | 4,25 (±0,62) | 4,05 (±0,75) | 4,20 (±0,74) |
| **Reader 3 (ML)** | 3,85 (±0,67) | 4,59 (±0,52) | 4,05 (±0,70) | 4,67 (±0,50) | 3,92 (±0,63) | 4,69 (±0,68) | 4,13 (±0,62) | 4,89 (±0,42) | 4,03 (±0,59) | 4,89 (±0,31) |
| **Delineation of cartilage/muscle–tendon/ligament–meniscus** | | | | | | | | | | |
| **Reader 1 (HA)** | 4,00 (±0,52) | 4,68 (±0,50) | 4,09 (±0,55) | 4,75 (±0,44) | 3,81 (±0,56) | 4,12 (±0,77) | 3,89 (±0,61) | 4,53 (±0,60) | 4,31 (±0,57) | 4,81 (±0,43) |
| **Reader 2 (KB)** | 3,79 (±0,55) | 4,15 (±0,71) | 3,96 (±0,56) | 4,28 (±0,61) | 4,00 (±0,59) | 4,17 (±0,69) | 4,11 (±0,65) | 4,25 (±0,64) | 4,07 (±0,74) | 4,24 (±0,65) |
| **Reader 3 (ML)** | 3,88 (±0,70) | 4,45 (±0,55) | 3,92 (±0,75) | 4,55 (±0,62) | 3,88 (±0,70) | 4,56 (±0,72) | 4,20 (±0,79) | 4,88 (±0,40) | 3,93 (±0,68) | 4,73 (±0,47) |
| **Fat saturation** | | | | | | | | | | |
| **Reader 1 (HA)** | 4,35 (±0,71) | 4,64 (±0,65) | 4,53 (±0,70) | 4,57 (±0,70) | 4,23 (±0,71) | 4,52 (±0,70) | – | – | – | – |
| **Reader 2 (KB)** | 4,08 (±0,49) | 4,04 (±0,53) | 4,15 (±0,59) | 4,03 (±0,54) | 4,07 (±0,53) | 4,15 (±0,59) | – | – | – | – |
| **Reader 3 (ML)** | 4,41 (±0,70) | 4,60 (±0,62) | 4,63 (±0,51) | 4,83 (±0,38) | 4,56 (±0,68) | 4,85 (±0,43) | – | – | – | – |
| **Movement artefacts** | | | | | | | | | | |
| **Reader 1 (HA)** | 3,97 (±0,57) | 4,97 (±0,16) | 4,60 (±0,79) | 4,96 (±0,26) | 3,65 (±0,74) | 3,61 (±0,66) | 4,69 (±0,66) | 4,63 (±0,63) | 4,95 (±0,23) | 4,95 (±0,23) |
| **Reader 2 (KB)** | 4,59 (±0,59) | 4,77 (±0,45) | 4,80 (±0,49) | 4,81 (±0,46) | 4,77 (±0,45) | 4,71 (±0,56) | 4,85 (±0,46) | 4,71 (±0,56) | 4,88 (±0,37) | 4,73 (±0,68) |
| **Reader 3 (ML)** | 4,16 (±0,72) | 4,88 (±0,37) | 4,07 (±0,58) | 4,61 (±0,54) | 4,11 (±0,67) | 4,61 (±0,74) | 4,55 (±0,64) | 4,61 (±0,50) | 4,60 (±0,55) | 4,61 (±0,23) |
| **Artefact foreign body** | | | | | | | | | | |
| **Reader 1 (HA)** | 4,57 (±0,65) | 4,86 (±0,38) | 4,64 (±0,63) | 4,88 (±0,35) | 0,84 (±1,79) | 4,86 (±0,38) | 4,93 (±0,27) | 5,00 (±0,00) | 4,62 (±0,65) | 5,00 (±0,00) |
| **Reader 2 (KB)** | 3.85 (±0.55) | 4,2 (±0,45) | 3,85 (±0,55) | 4 (±0) | 3.85 (±0.69) | 4 (±0) | 4.85 (±0.38) | 4.4 (±0.55) | 4.15 (±0.55) | 4.8 (±0.45) |
| **Reader 3 (ML)** | 4,33 (±0,89) | 4,75 (±0,50) | 4,30 (±0,82) | 4,75 (±0,50) | 3,92 (±0,90) | 4,50 (±0,58) | 4,50 (±0,71) | 5,00 (±0,00) | 4,33 (±0,71) | 4,75 (±0,50) |

Supplement 1 - Rater-specific qualitative ratings (mean ± SD) comparing sMRI and DL-MRI across sequences (PD-FS coronal/sagittal/axial, T2, T1) for overall quality, anatomical delineation, fat saturation, motion artefacts, and foreign-body artefacts.
